# Multicenter evaluation of Coyote FlashDetect LyocartE multiplex assays for six respiratory pathogens: a comparative analysis with Sansure and Cepheid assays

**DOI:** 10.1128/spectrum.03236-25

**Published:** 2026-09-17

**Authors:** Cong Liu, Lei Feng, Gaowei Fan, Huiying Lai, Linlin Xiao, Yanxi Han, Duo Wang, Wangyu Feng, Yingshuo Ma, Ziqiang Li, Qiang Zhang, Jinming Li, Rui Zhang

**Affiliations:** 1 National Center for Clinical Laboratories, Institute of Geriatric Medicine, Chinese Academy of Medical Sciences, Beijing Hospital/ National Center of Gerontology 731304, Beijing, P. R. China; 2 National Center for Clinical Laboratories, Chinese Academy of Medical Sciences & Peking Union Medical College, Beijing, P. R. China; 3 Beijing Engineering Research Center of Laboratory Medicine, Beijing Hospital 117555, Beijing, P. R. China; 4 Department of Clinical Laboratory, Beijing Chao-Yang Hospital, Capital Medical University 12517, Beijing, P. R. China; 5 Beijing Center for Clinical Laboratory, Beijing, P. R. China; 6 Department of Clinical Laboratory, Beijing Hospital, National Center of Gerontology Institute of Geriatric Medicine, Chinese Academy of Medical Sciences, Beijing, P. R. China; 7 Department of Laboratory Medicine, Shanghai Jiaotong University Affiliated Sixth People's Hospital South Campus, Shanghai, P. R. China; 8 Department of Clinical Laboratory, Beijing Shunyi Hospital 849747 , Beijing, P. R. China; Houston Methodist Hospital, Houston, Texas, USA

**Keywords:** Cepheid Xpert Xpress assay, Coyote FlashDetect LyocartE assay, multicenter evaluation, multiplex mPOCT, six respiratory pathogens

## Abstract

Respiratory diseases caused by common respiratory pathogens, such as influenza A/B (Flu A/B), severe acute respiratory syndrome coronavirus-2 (SARS-CoV-2), respiratory syncytial virus (RSV), adenovirus (ADV), and *Mycoplasma pneumoniae* (MP), share many of the same symptoms and may progress to severe complications in some cases. Multiplex molecular point-of-care testing (mPOCT) enables automated and rapid differential diagnosis for timely intervention. This study independently evaluated the clinical performance of Coyote FlashDetect LyocartE SARS-CoV-2/FluA/FluB and FlashDetect LyocartE RSV/MP/ADV multiplex mPOCT assays using the Sansure as the primary comparator, followed by a subset-based, target-specific paired comparison with Cepheid mPOCT assay for selected respiratory pathogens (FluA, FluB, and RSV). Specifically, 642 and 427 throat swab specimens were used to evaluate the Coyote SARS-CoV-2/FluA/FluB and Coyote RSV/MP/ADV multiplex mPOCT assays against Sansure assays, respectively. Coyote assays demonstrated high concordance with Sansure assays, with overall percent agreement (OPA) values ranging from 93.8% to 100.0% for six pathogens covered by two Coyote assays. Most discrepancies with Sansure assays occurred in samples with cycle threshold (Ct) values ≥35, especially among FluA samples. Moreover, 414, 414, and 215 specimens were allocated for FluA, FluB, and RSV, respectively, to conduct target-specific paired comparison between Coyote and Cepheid. Each Coyote assay also demonstrated high concordance with the Cepheid assays, with PPA values of 96.0%, 100.0%, and 98.3% for FluA, FluB, and RSV, respectively. These results support the use of Coyote mPOCT assays in specific settings. Coyote assays demonstrated a high degree of concordance for shared targets with comparator methods, supporting their use as a rapid and user-friendly diagnostic approach for multiplex respiratory pathogen detection.

**IMPORTANCE:**

This study highlights the value of multiplex molecular point-of-care tests. The POCT assays demonstrate high concordance with the laboratory-based lab tests in this retrospective specimen cohort, offering a viable, rapid, and user-friendly alternative for multiplex respiratory pathogen detection and hold the promise to be used in primary healthcare settings and public health surveillance.

## INTRODUCTION

Acute respiratory infections (ARIs) are the fourth leading cause of death worldwide, causing ~2.5 million deaths each year (1, 2). Acute upper respiratory infections (AURIs) are usually mild to moderate and self-limited, with viruses accounting for ~85% of cases (3). However, without timely diagnosis and intervention, some viruses may progress to the lower respiratory tract, leading to severe complications such as pneumonia, respiratory failure, and superimposed bacterial infection (4–6). The five respiratory viruses including influenza A virus (Flu A), influenza B virus (Flu B), severe acute respiratory syndrome coronavirus-2 (SARS-CoV-2), respiratory syncytial virus (RSV), and adenovirus (ADV), together with the atypical bacterial *Mycoplasma pneumoniae* (MP), are considered high-priority targets, characterized by the tendency to cause severe complications, high prevalence, and availability of specific therapies (7–11). Flu A, Flu B, and SARS-CoV-2 share highly similar clinical symptoms but require different treatments (12), while RSV, ADV, and MP are major causes of severe complications in children (13–15). Furthermore, coinfections from these pathogens occur in approximately 10% of ARIs and are associated with worse clinical outcomes (16–20). Therefore, reliable multiplex diagnostic assays are crucial to distinguish between these pathogens timely and accurately, which has important implications for both effective treatment and prognostic evaluation.

Nucleic acid amplification tests (NAATs) have been widely used for the clinical detection of infectious pathogens due to their high sensitivity and specificity. Among them, real-time polymerase chain reaction (PCR) is the gold standard for virologic diagnosis (21, 22). Even though widely applied, conventional laboratory-based real-time PCR requires manual sample preprocessing and nucleic extraction, necessitating trained personnel and posing a risk of cross-contamination (23, 24). Also, it should be performed in a standardized molecular laboratory with clearly defined functional areas. In addition, the overall turnaround time (TAT) of 2.5 to 4 h limits its applicability in urgent diagnostic scenarios (25, 26). These limitations hinder the implementation of conventional real-time PCR in community and primary healthcare settings.

Recently, the advent of point-of-care molecular testing (mPOCT) platforms has enabled automatic detection, shorter TAT, and deployment in resource-constrained settings, overcoming the limitations of conventional laboratory-based real-time PCR and supporting continuous random-access testing capability (27). Currently, automatic cartridge-based mPOCT assays, the Coyote FlashDetect LyocartE SARS-CoV-2/FluA/FluB and Coyote FlashDetect LyocartE RSV/MP/ADV, have been approved by the National Medical Products Administration (NMPA) in China. However, its clinical performance remains unclear. Here, we conducted a multicenter evaluation of the Coyote FlashDetect LyocartE multiplex mPOCT assays using the conventional Sansure laboratory-based real-time PCR assay as the primary comparator, as well as a subset-based, target-specific paired comparison between Coyote and Cepheid mPOCT platforms for selected respiratory pathogens.

## MATERIALS AND METHODS

### Clinical specimens

Residual throat swab (TS) specimens with positive clinical standard of care (SOC) testing results for at least one of six target pathogens (Flu A, Flu B, SARS-CoV-2, RSV, ADV, or MP), along with specimens with negative SOC testing results for all six pathogens, were retrospectively collected between November 2023 and May 2024 and between November 2024 and April 2025 from four clinical centers representing three different levels of medical institutions in China: Affiliated Beijing Chaoyang Hospital of Capital Medical University (comprehensive tertiary hospital), Beijing Hospital (geriatric hospital), Beijing Shunyi Hospital (primary hospital), and Shanghai Fengxian Central Hospital (primary hospital). Although the SOC-based retrospective specimen selection strategy enabled sufficient target-positive specimens for statistical evaluation, the selected specimens did not represent a consecutive and unbiased series of all specimens received during the study period. SOC testing protocols at the participating clinical centers were summarized in Table 1. The National Center for Clinical Laboratories (NCCL) served as the central laboratory for testing, evaluation, and comparison.

**TABLE 1:** Standard of care (SOC) testing protocols at the participating clinical centers

Clinical sites	Standard of care tests	Manufacturers
Beijing Hospital	Mycoplasma pneumoniae/ Chlamydia pneumoniae / Adenovirus nucleic acid detection kit (PCR-Fluorescence Probing)	Autobio-diagnostics Co., Ltd., Zhengzhou, China
	Influenza A virus/Influenza B virus/ Respiratory syncytial virus nucleic acid detection kit (PCR-Fluorescence Probing)	Autobio-diagnostics Co., Ltd., Zhengzhou, China
	Diagnostic Kit for Novel-Coronavirus (2019-nCoV) RNA (Isothermal Amplification-Real Time Fluorescence Assay)	Ustar Biotech Co., Ltd., Hangzhou, China
Beijing Chaoyang Hospital	Six Respiratory Pathogens Nucleic Acid Diagnostic Kit (PCR-Fluorescence Probing)	Sansure Biotech Co., Ltd., Changsha, China
	Multiple Real-Time PCR Kit for Detection of 2019-nCoV (PCR-Fluorescence Probing)	Applied Biological Technologies Co., Ltd., Beijing, China
Beijing Shunyi Hospital	Multiplex Real-Time PCR Diagnostic Kit for Rapid Detection of Six Respiratory Viruses (PCR-Fluorescence Probing)	Applied Biological Technologies Co., Ltd., Beijing, China
	Nucleic Acid Test Kit COVID-19 (SARS-CoV-2) (PCR-Fluorescence Probing)	Easy Diagnosis Biomedicine Co., Ltd., Wuhan, China
	Mycoplasma pneumoniae DNA Diagnostic Kit (Fluorescence PCR)	Abiores Technology Co., Ltd., Beijing, China
Shanghai Fenxian Central Hospital	FlashDetect LyocartE SARS-CoV-2&Flu A&Flu B Assay (PCR-Fluorescence Probing)	Coyote Bioscience Co., Ltd., Yixing, China
	Multiplex Real-Time PCR Diagnostic Kit for Rapid Detection of Six Respiratory Viruses (PCR-Fluorescence Probing)	Applied Biological Technologies Co., Ltd., Beijing, China
	Diagnostic Kit for Mycoplasma pneumoniae DNA (PCR-Fluorescence Probing) Diagnostic Kit for Respiratory Syncytial Virus RNA (PCR-Fluorescence Probing) Diagnostic Kit for adenovirus DNA (PCR-Fluorescence Probing)	DAAN Gene Co., Ltd., Guangzhou, China

The Sansure conventional laboratory-based real-time PCR assays were designed as the primary comparator method in this study, given their proven high performance and their use as a reference standard or orthogonal assay in previous studies (28–33). Based on the Sansure results, 289, 274, 63, 138, 91, and 139 specimens were classified as positive for SARS-CoV-2, FluA, FluB, RSV, ADV, and MP, respectively, as well as 77 specimens classified as negative. Notably, 131 specimens (14.0%) were classified as coinfections. The most prevalent co-infection was FluA/SARS-CoV-2 (5.8%, 54/933), followed by FluA/MP (2.6%, 24/933) and MP/ADV (1.5%, 14/933).

Demographic and limited clinical data were collected for comprehensive analysis, including the date of specimen collection, sex, age, patient type. After the clinical SOC diagnostic testing, the remnant specimens were archived frozen at −80℃ immediately until transporting to the central laboratory.

### Study design

**Fig 1:**
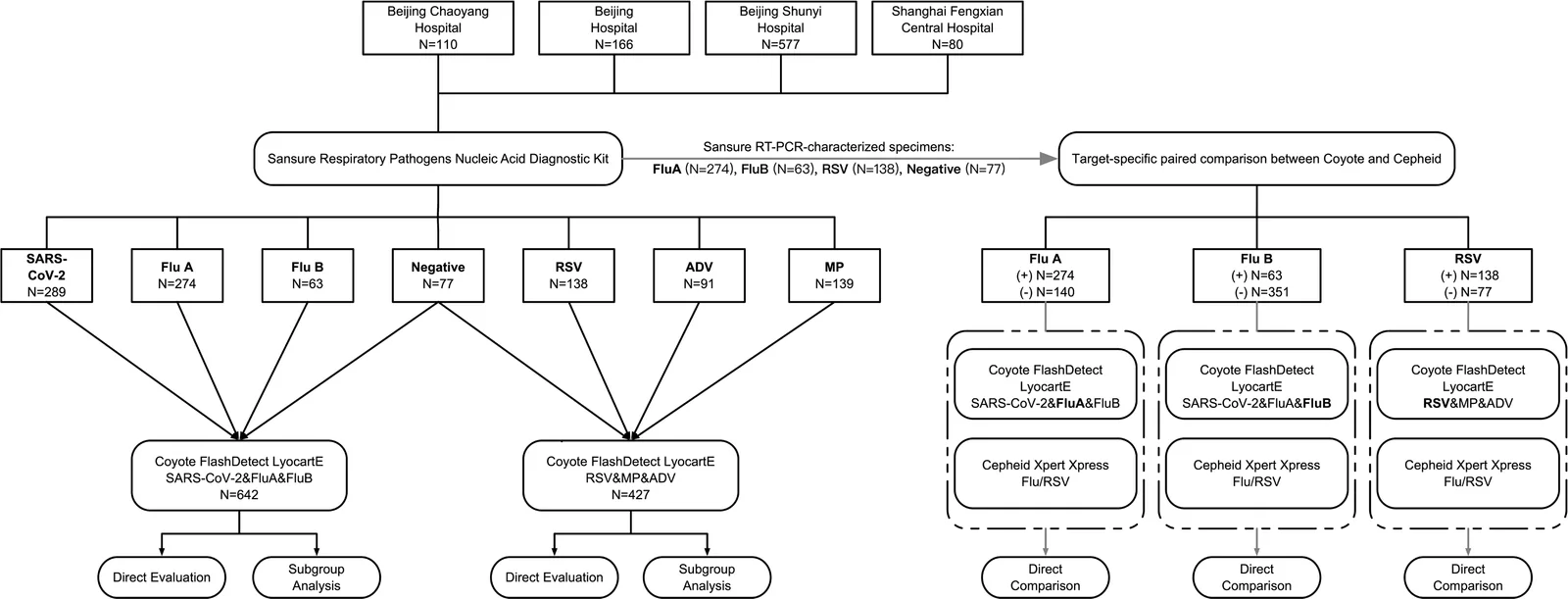
Study design and evaluation workflow. Coinfections were present among the specimens, so the total number of specimens for a given assay was lower than the sum of the individual specimens included.

To thoroughly evaluate the Coyote mPOCT assays, a two-step framework was implemented, comprising a clinical performance evaluation of the Coyote FlashDetect LyocartE multiplex mPOCT assays using the Sansure conventional laboratory-based RT-PCR assay as the primary comparator, followed by a subset-based, target-specific paired comparison with the Cepheid mPOCT assays for FluA, FluB, and RSV using pre-allocated specimens. It should be noted that not all specimens were tested on all three platforms; therefore, no complete head-to-head comparison across all assays was performed (Fig. 1).

First, the overall performance, as well as subgroup analyses across patient types and clinical sites, of the investigational Coyote mPOCT assays were separately evaluated against Sansure. Specifically, 642 Sansure-classified specimens were available for performance evaluation of Coyote FlashDetect LyocartE SARS-CoV-2/FluA/FluB assay, including SARS-CoV-2-positive (*N* = 289), FluA-positive (*N* = 274), FluB-positive (*N* = 63), and negative (*N* = 77) specimens. For the Coyote FlashDetect LyocartE RSV/MP/ADV assay, 427 specimens were included, comprising RSV-positive (*N* = 138), ADV-positive (*N* = 91), MP-positive (*N* = 139), and negative (*N* = 77) specimens.

Second, the target-specific paired comparison was performed between Coyote and Cepheid assays with a subset of specimens specifically allocated for FluA, FluB, and RSV target evaluation. Specifically, 414 Sansure-classified specimens (274 positive and 140 negative for FluA) were included for target-specific performance evaluation of FluA, and 414 Sansure-classified specimens (63 positive and 351 negative for FluB) were included for target-specific performance evaluation of FluB. For each target, the comparative performance between the Coyote FlashDetect LyocartE SARS-CoV-2/FluA/FluB assay and the Cepheid Xpert Xpress Flu/RSV assay was assessed. In parallel, 215 Sansure-classified specimens (138 positive and 77 negative for RSV) were included for target-specific comparative evaluation of RSV detection between the Coyote FlashDetect LyocartE RSV/MP/ADV assay and the Cepheid Xpert Xpress Flu/RSV assay.

### Result interpretation

All performance estimates reported herein are conditional on each comparator method. Result pairs were classified as concordant positive (Coyote positive/comparator positive), concordant negative (Coyote negative/comparator negative), positive discordant (Coyote positive/comparator negative), or negative discordant (Coyote negative/comparator positive), without assuming clinical truth in the absence of an established reference standard. Discordant results were not adjudicated by an independent reference method or a third testing platform.

### Coyote FlashDetect LyocartE assays

**Fig 2:**
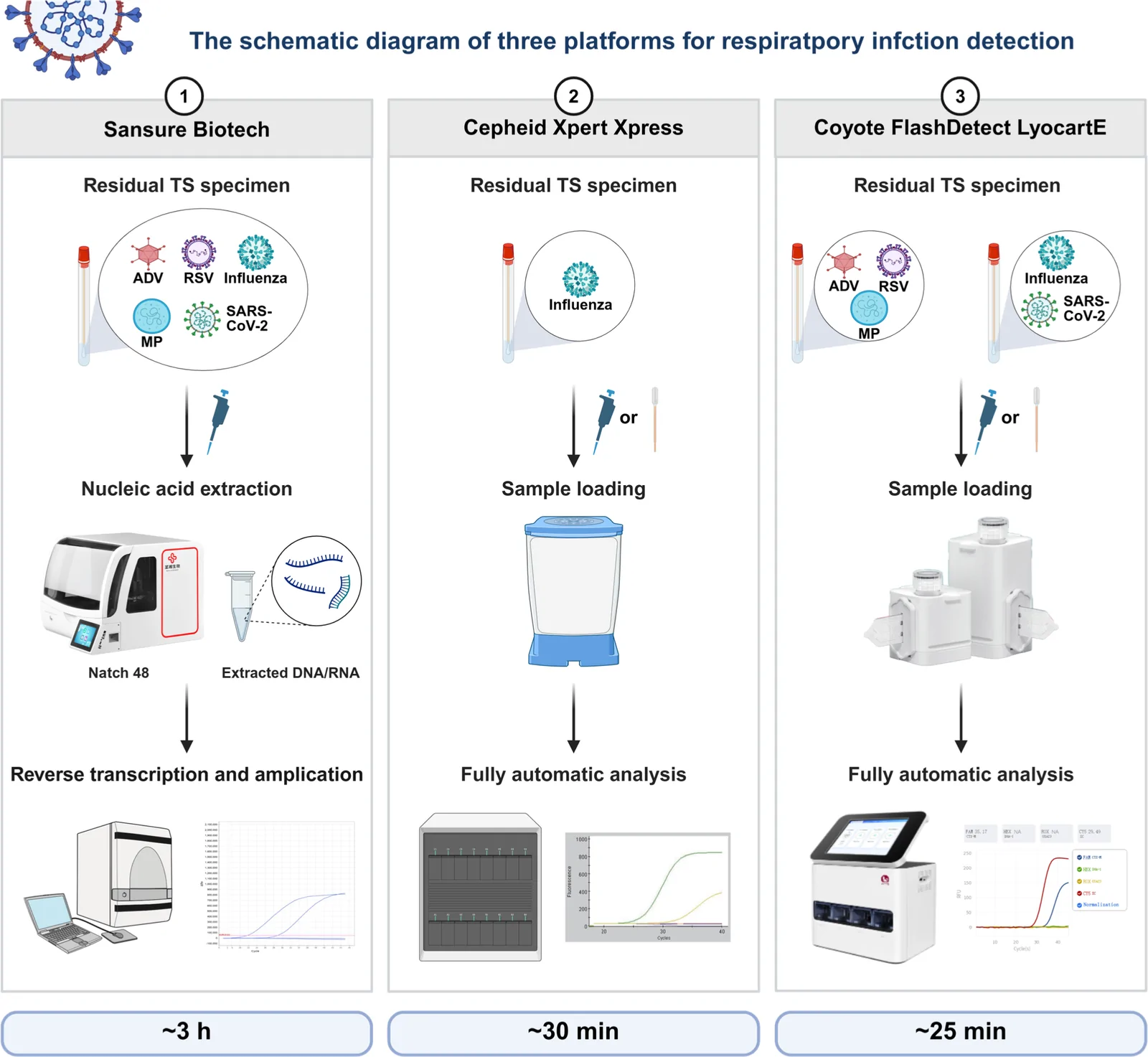
The schematic diagrams of three platforms for respiratory infection detection. Created in BioRender. Zhang, R. (2025) https://BioRender.com/l9klvny.

Coyote SARS-CoV-2/FluA/FluB and Coyote RSV/MP/ADV assays are also automated and cartridge-based multiplex real-time PCR assays. Two cartridges are included: one targets SARS-CoV-2, FluA, and FluB, while the other targets RSV, MP, and ADV. All tests were performed on the FlashDetect Flash10 system according to the manufacturer’s manuals. Briefly, 400 μL and 350 μL of the sample were added to the sample chambers of the SARS-CoV-2/FluA/FluB cartridge and RSV/MP/ADV cartridge, respectively. All subsequent steps including sample preparation, nucleic acid extraction, reverse transcription (for RNA viruses), and nucleic acid amplification were performed automatically, and the final result was displayed on the screen within approximately 25 min after loading the cartridge (Fig. 2).

### Comparator testing

The Sansure Respiratory Pathogens Nucleic Acid Diagnostic assays targeting FluA, FluB, RSV, ADV, MP, and SARS-CoV-2 are based on conventional laboratory-based real-time PCR. Nucleic acids were extracted and purified from 200 μL of specimen using the automated Sansure Natch Nucleic Acid Extraction System (Sansure Biotech). The detection of respiratory pathogens was then conducted on the Applied Biosystems 7500 Fast Real-Time PCR System (Thermo Fisher Scientific). All procedures, including nucleic acid extraction, real-time PCR, and result interpretation, were performed according to the manufacturer’s instructions (Fig. 2).

Cepheid Xpert Xpress Flu/RSV assay is a rapid, automated, and cartridge-based multiplex real-time PCR assay for simultaneous detection and differentiation of FluA, FluB, and RSV. According to the manufacturer’s instructions, 300 μL of the sample was added to the sample chamber of the cartridge. Then, the cartridge was loaded into the GeneXpert Xpress system following the system instructions on the screen. All subsequent steps including sample preparation, nucleic acid extraction, reverse transcription, and nucleic acid amplification were performed automatically, and the final results were displayed on the screen within approximately 30 min after loading the cartridge (Fig. 2).

### Statistical analysis

For performance evaluation of each multiplex mPOCT assay, all target-specific results generated from each specimen were incorporated into the statistical analysis. The agreement between assays was evaluated based on qualitative results using the overall percent agreement (OPA), positive percent agreement (PPA), and negative percent agreement (NPA), which were calculated as the ratios of concordant results to total results among all, positive and negative samples determined by comparator methods, respectively. The 95% confidence intervals (*CIs*) for OPA, PPA, and NPA were calculated using the Wilson score method. Cohen’s *κ* values were also used to assess the level of agreement between two detection methods. In addition, the cycle threshold (Ct) values of the positive samples obtained from the Sansure assay, the Cepheid Xpert Xpress Flu/RSV assay, and the Coyote FlashDetect LyocartE assays were analyzed. The correlations of Ct values between assays were evaluated by linear regression using Pearson’s correlation coefficient (*r*), and average Ct values were compared using the paired *t*-test. A *P* value > 0.05 was considered no significance (n.s.), and significance levels were denoted as follows: *P < 0.05, **P < 0.01, and ***P < 0.001. Statistical analyses and plots were performed using MedCalc statistical software version 20.027 (MedCalc Software Ltd., Ostend, Belgium) and ggplot2 package (v3.5.1) in R version 4.4.2 (R Foundation for Statistical Computing, Vienna, Austria).

## RESULTS

### Subject demographics and sample characteristics

A total of 933 TS specimens were collected from four clinical centers in China. These samples were used to evaluate the Coyote SARS-CoV-2/FluA/FluB (*N* = 642) and RSV/MP/ADV (*N* = 427) assays, as well as conduct subset-based, target-specific paired comparison between Coyote and Cepheid. The demographic characteristics of the study population for each assay were summarized in Table 2. Gender distribution was generally balanced in both the overall specimen set and the assay-specific subsets. The age distribution included pediatric patients less than 18 years of age (*N* = 370, 39.7%), adults aged 18–65 years (*N* = 390, 41.8%), and adults older than 65 years of age (*N* = 173, 18.5%). Notably, the majority of specimens for Coyote RSV/MP/ADV were pediatric patients less than 18 years of age (*N* = 301, 70.5%). Additionally, the majority of specimens were collected from outpatients (*N* = 417, 44.7%), followed by 30.4% from the emergency department (*N* = 284) and 24.9% from inpatients (*N* = 232).

**TABLE 2:** Demographics and specimen information

Category	Total	Coyote SARS-CoV-2/FluA/FluB	Coyote RSV/MP/ADV
Total patients, *N*	933	642	427
Age (years)			
Median (min-max)	31 (0–95)	37 (0–95)	8 (0–95)
Mean ± SD	33.5 ± 27.1	40.9 ± 25.6	21.0 ± 25.6
Age < 18, *N* (%)	370 (39.7%)	144 (22.4%)	301 (70.5%)
Age ≥ 65, *N* (%)	173 (18.5%)	150 (23.4%)	51 (11.9%)
Sex, *N* **(%)**			
Male	443 (47.5%)	310 (48.3%)	197 (46.1%)
Female	490 (52.5%)	332 (51.7%)	230 (53.9%)
Study site, *N* **(%)**			
Chaoyang District, Beijing	110 (11.8%)	83 (12.9%)	69 (16.2%)
Shunyi District, Beijing	577 (61.8%)	349 (54.4%)	300 (70.3%)
Dongcheng District, Beijing	166 (17.8%)	147 (22.9%)	33 (7.7%)
Fengxian District, Shanghai	80 (8.6%)	63 (9.8%)	25 (5.9%)
Patients type, *N* **(%)**			
Inpatient	232 (24.9%)	155 (24.1%)	121 (28.3%)
Outpatient	417 (44.7%)	231 (36.0%)	255 (59.7%)
Emergency department	284 (30.4%)	256 (39.9%)	51 (11.9%)

### The agreement analysis between Coyote multiplex mPOCT assays and conventional laboratory-based real-time PCR assay

**Fig 3:**
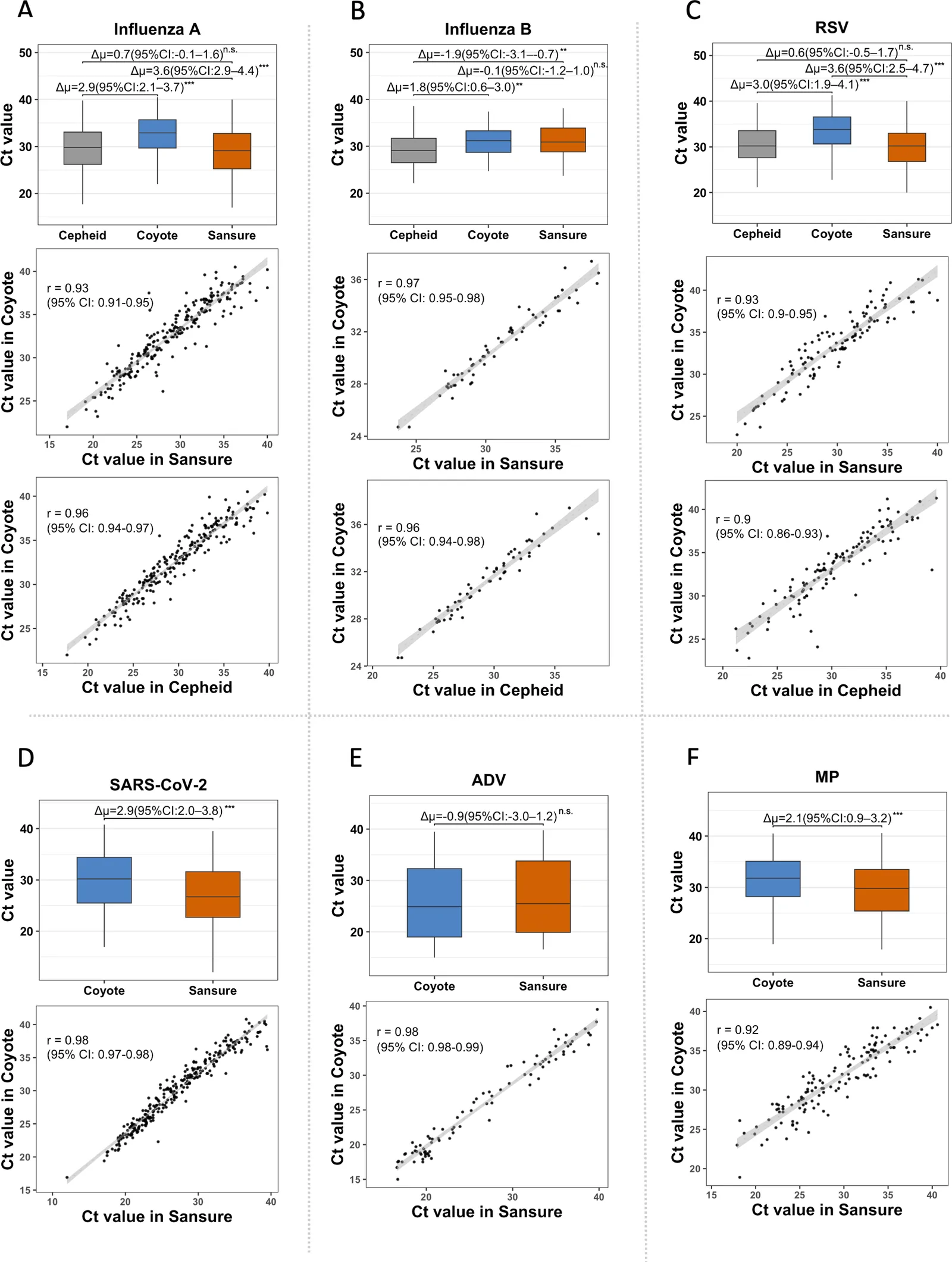
Distribution and inter-assay correlation of Ct values obtained from Coyote FlashDetect LyocartE, Cepheid Xpert Xpress, and Sansure assays for six respiratory pathogens. Panels show individual pathogens: (**A**) influenza A virus, (**B**) influenza B virus, (**C**) respiratory syncytial virus, (**D**) SARS-CoV-2, (**E**) adenovirus, and (**F**) *Mycoplasma pneumoniae*. Box-and-whisker plots show the medians (center lines within each box), the 25th and 75th percentiles (bottom and top edges of each box), and the range from the 5th to 95th percentiles (whiskers). Differences in mean Ct values (Δ*μ*) between groups and their 95% confidence intervals (95% CI) are indicated. Statistical significance is denoted as n.s. (no significance, *P* > 0.05), *P < 0.05, **P < 0.01, and ***P < 0.001. Scatter plots show the correlation of Ct values between two assays for the six pathogens. The Pearson correlation coefficient (*R*) and 95% confidence interval (95% CI) are labeled.

The clinical performance of the two Coyote multiplex mPOCT assays, including FlashDetect LyocartE SARS-CoV-2/FluA/FluB assay and FlashDetect LyocartE RSV/MP/ADV assay, was first assessed against the Sansure real-time PCR assay (Table 3). Overall, the results demonstrated high concordance for each of the six targets. Specifically, for the Coyote FlashDetect LyocartE SARS-CoV-2/FluA/FluB assay, the OPA was 96.7% (621/642; 95% CI: 95.0% to 98.0%) for SARS-CoV-2, 93.8% (602/642; 95% CI: 91.6% to 95.5%) for FluA, and 100.0% (642/642; 95% CI: 99.4% to 100.0%) for FluB. Parallelly, for the Coyote FlashDetect LyocartE RSV/MP/ADV assay, OPAs for RSV, MP, and ADV were 97.9% (418/427; 95%CI: 96.0% to 99.0%), 98.4% (420/427; 95% CI: 96.7% to 99.3%), and 95.3% (407/427; 95% CI: 92.9% to 97.1%), respectively. Additionally, the NPA values were above 94.6% for each of the six targets. However, as for PPA, values for five of the six pathogens were above 94.2%, with the exception of FluA, which showed a slightly lower value of 88.0% (95% CI: 83.5% to 91.6%). The results of the Ct-stratified agreement analysis demonstrated that PPA values were above 98.1% for each of the six targets among samples with high viral loads (Ct < 35), whereas markedly reduced PPA values were observed in samples with higher Ct values, ranging from 50% to 100% (Table 4). For positive samples detected by both assays, a strong correlation in Ct values was observed, with Pearson’s *r* values exceeding 0.90 for each of the six pathogens (Fig. 3).

**TABLE 3:** The agreement analysis between the Coyote FlashDetect LyocartE assays and the Sansure assay^*a*^

Coyote FlashDetect LyocartE assays			Sansure Biotech		OPA, *n/N* (95% CI)	PPA, *n/N* (95% CI)	NPA, *n/N* (95% CI)	Cohen’s *κ* (95% CI)
			Positive	Negative				
Coyote FlashDetect SARS-CoV-2/FluA/FluB	SARS- CoV-2	Positive	275	7	96.7%, 621/642 (95.0%–98.0%)	95.2%, 275/289 (92.0%–97.3%)	98.0%,346/353 (96.0%–99.2%)	0.93 (0.91–0.96)
		Negative	14	346				
		Total	289	353				
	FluA	Positive	241	7	93.8%, 602/642 (91.6%–95.5%)	88.0%, 241/274 (83.5%–91.6%)	98.1%, 361/368 (96.1%–99.2%)	0.87 (0.83–0.91)
		Negative	33	361				
		Total	274	368				
	FluB	Positive	63	0	100.0%, 642/642 (99.4%–100.0%)	100.0%, 63/63 (94.3%–100.0%)	100.0%, 579/579 (99.4%–100.0%)	1.00 (1.00–1.00)
		Negative	0	579				
		Total	63	579				
Coyote FlashDetect RSV/MP/ADV	RSV	Positive	130	1	97.9%, 418/427 (96.0%–99.0%)	94.2%, 130/138 (88.9%–97.5%)	99.7%, 288/289 (98.1%–100.0%)	0.95 (0.92–0.98)
		Negative	8	288				
		Total	138	289				
	MP	Positive	137	5	98.4%, 420/427 (96.7%–99.3%)	98.6%, 137/139 (94.9%–99.8%)	98.3%, 283/288 (96.0%–99.4%)	0.96 (0.94–0.99)
		Negative	2	283				
		Total	139	288				
	ADV	Positive	89	18	95.3%, 407/427 (92.9%–97.1%)	97.8, 89/91 (92.3%–99.7%)	94.6, 318/336 (91.7%–96.8%)	0.87 (0.81–0.92)
		Negative	2	318				
		Total	91	336				

^*a*^ The agreement parameters of the Coyote FlashDetect LyocartE assays relative to conventional Sansure real-time PCR assay are displayed by pathogen type, including overall percent agreement (OPA), positive percent agreement (PPA), negative percent agreement (NPA) with 95% confidence intervals (95% CIs) and Cohen’s *κ* values. *n/N* = number of concordant results/total number of samples.

**TABLE 4:** Ct-stratified agreement analysis between Coyote FlashDetect LyocartE mPOCT and comparator methods

Target pathogen	Comparator method	Ct stratum	Sample number	Discordant number	PPA, (95% CI)
FluA	Sansure RT-PCR	Ct < 35	216	4	98.1%, (95.3%–99.5%)
		Ct ≥ 35	58	29	50.0%, (36.6%–63.4%)
FluB	Sansure RT-PCR	Ct < 35	49	0	100.0%, (92.7%–100.0%)
		Ct ≥ 35	14	0	100.0%, (76.8%–100.0%)
SARS-CoV-2	Sansure RT-PCR	Ct < 35	246	2	99.2%, (97.1%–99.9%)
		Ct ≥ 35	43	12	72.1%, (56.3%–84.7%)
RSV	Sansure RT-PCR	Ct < 35	109	1	99.1%, (95.0%–100.0%)
		Ct ≥ 35	29	7	75.9%, (56.5%–89.7%)
ADV	Sansure RT-PCR	Ct < 35	71	0	100.0%, (94.9%-100.0%)
		Ct ≥ 35	20	2	90.0%, (68.3%–98.8%)
MP	Sansure RT-PCR	Ct < 35	113	0	100.0%, (96.8%–100.0%)
		Ct ≥ 35	26	2	92.3%, (74.9%–99.1%)
FluA	Cepheid Xpert Xpress	Ct < 35	205	2	99.0%, (96.5%–99.9%)
		Ct ≥ 35	43	8	81.4%, (66.6%–91.6%)
FluB	Cepheid Xpert Xpress	Ct < 35	58	0	100.0%, (93.8%–100.0%)
		Ct ≥ 35	3	0	100.0%, (29.2%–100.0%)
RSV	Cepheid Xpert Xpress	Ct < 35	96	0	100.0%, (96.2%–100.0%)
		Ct ≥ 35	21	2	90.5%, (69.6%–98.8%)

**Fig 4:**
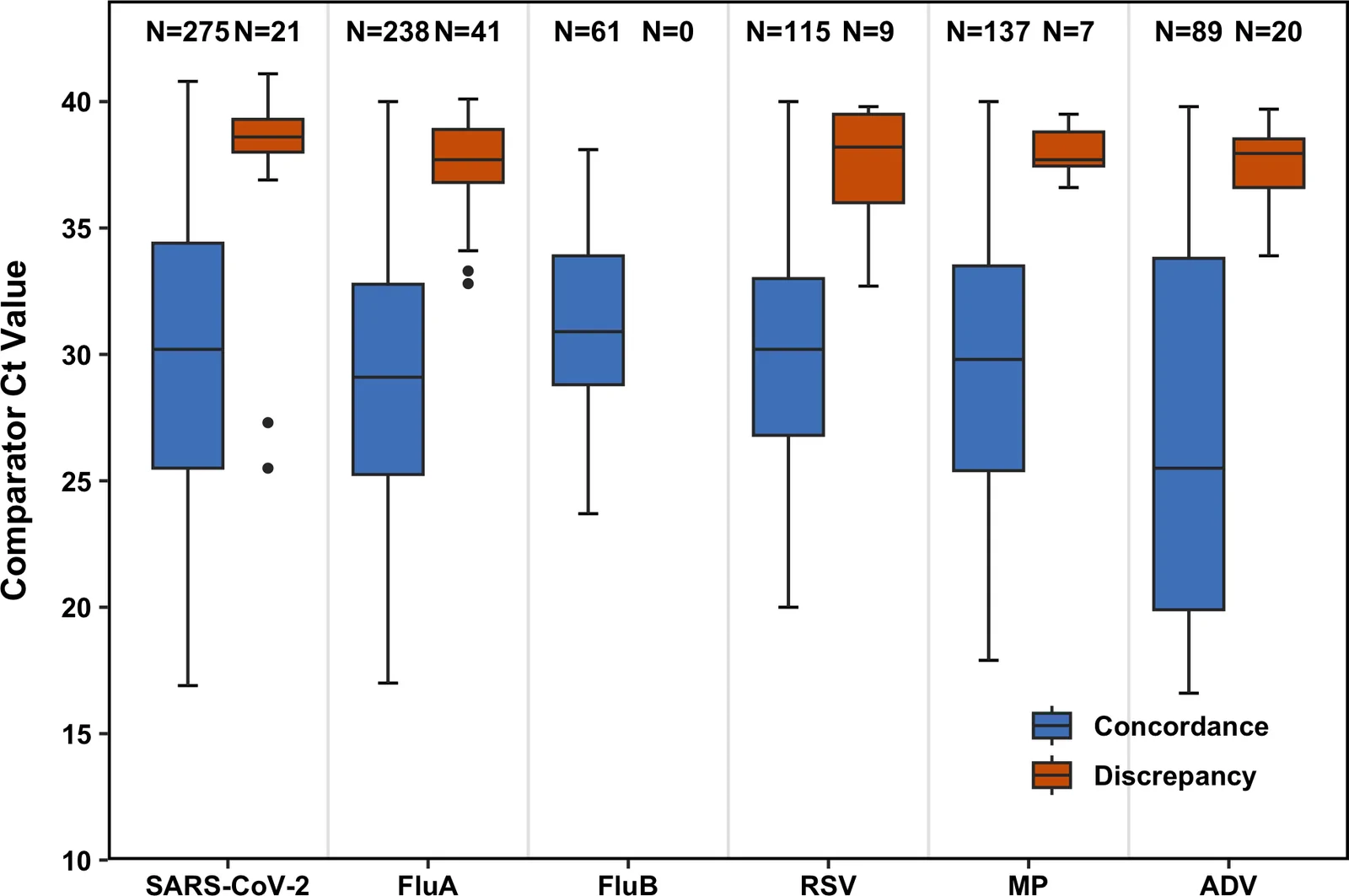
Distribution of comparator Ct values for concordant and discrepant results between Sansure and Coyote. *N* represents sample number. Box-and-whisker plots show the medians (center lines within each box), the 25th and 75th percentiles (bottom and top edges of each box), and the range from the 5th to 95th percentiles (whiskers). Outliers are shown as black dots beyond the 5th to 95th percentiles. Comparator Ct values were reported by the Sansure assay or the Coyote assay when the Sansure assay produced negative results.

Overall, 98 discordant results were identified between Coyote and Sansure, comprising 39 positive discordant and 59 negative discordant results. Among the positive discordant specimens (8 FluA, 1 RSV, 7 SARS-CoV-2, 5 MP, and 18 ADV), 94.9% (37/39) of samples were characterized with high Ct values. In terms of negative discordant specimens, FluA accounted for the highest proportion (55.9%, 33/59), followed by 14 SARS-CoV-2, 8 RSV, 2 MP, and 2 ADV. Notably, only seven negative discordant specimens exhibited Ct values ≤ 35, including four FluA cases (Ct = 32.8, 33.3, 34.1, and 34.7), two SARS-CoV-2 cases (Ct = 25.5 and 27.3), and one RSV case (Ct = 30.5) (Table 4). The distribution of Ct values for each target with concordant and discrepant results was displayed in Fig. 4.

### The agreement analysis between Coyote multiplex mPOCT assays and Sansure across different patient types

To explore the clinical performance of Coyote mPOCT assays for each target pathogen across different patient types, specimens were categorized into inpatient, outpatient, and emergency department (ED) groups (Table 5). In general, the results demonstrated high concordance across patient types for each of the six targets against the Sansure.

**TABLE 5:** The agreement analysis between Coyote multiplex mPOCT assays and Sansure assay across different patient types^*b*^

mPOCT assays	Target pathogens	Patients types	OPA, *n*/*N* (95% CI)	PPA, *n*/*N* (95% CI)	NPA, *n*/*N* (95% CI)	Cohen’s *κ* (95% CI)
Coyote FlashDetect SARS-CoV-2/FluA/FluB	SARS-CoV-2	Inpatient	96.8%, 150/155 (92.6%–98.9%)	93.3%, 70/75 (85.1%–97.8%)	100.0%, 80/80 (95.5%–100.0%)	0.94 (0.88–0.99)
		Outpatient	97.8%, 226/231 (95.0%–99.3%)	96.2%, 100/104 (90.4%–98.9%)	99.2%, 126/127 (95.7%–100.0%)	0.96 (0.92–0.99)
		Emergency department	95.7%, 245/256 (92.4%–97.8%)	95.5%, 105/110 (89.7%–98.5%)	95.9%, 140/146 (91.3%–98.5%)	0.91 (0.86–0.96)
	FluA	Inpatient	93.5%, 145/155 (88.5%–96.9%)	84.8%, 56/66 (73.9%–92.5%)	100.0%, 89/89 (95.9%–100.0%)	0.87 (0.79–0.95)
		Outpatient	94.8%, 219/231 (91.1%–97.3%)	89.4%, 84/94 (81.3%–94.8%)	98.5%, 135/137 (94.8%–99.8%)	0.89 (0.83–0.95)
		Emergency department	93.4%, 239/256 (89.6%–96.1%)	89.5%, 102/114 (82.3%–94.4%)	96.5%, 137/142 (92.0%–98.8%)	0.86 (0.80–0.93)
	FluB	Inpatient	100%, 155/155 (97.6%–100.0%)	100.0%, 1/1 (2.5%–100.0%)	100.0%, 154/154 (97.6%–100.0%)	1.00 (1.00–1.00)
		Outpatient	100%, 231/231 (98.4%–100.0)	100%, 14/14 (76.8%–100.0%)	100.0%, 217/217 (98.3%–100.0%)	1.00 (1.00–1.00)
		Emergency department	100%, 256/256 (98.6%–100.0%)	100%, 48/48 (92.6%–100.0%)	100.0%, 208/208 (98.2%–100.0%)	1.00 (1.00–1.00)
Coyote FlashDetect RSV/MP/ADV	RSV	Inpatient	95.9%, 116/121 (90.6%–98.6%)	93.1%, 54/58 (83.3%–98.1%)	98.4%, 62/63 (91.5%–100.0%)	0.92 (0.85–0.99)
		Outpatient	98.8%, 252/255 (96.6%–99.8%)	94.9%, 56/59 (85.9%–98.9%)	100.0%, 196/196 (98.1%–100.0%)	0.97 (0.93–1.00)
		Emergency department	98.0%, 50/51 (89.6%–100.0)	95.2%, 20/21 (76.2%–99.9%)	100.0%, 30/30 (88.4%–100.0%)	0.96 (0.88–1.00)
	MP	Inpatient	99.2%, 120/121 (95.5%–100.0%)	95.0%, 19/20 (75.1%–99.9%)	100.0%, 101/101 (96.4%–100.0%)	0.97 (0.91–1.00)
		Outpatient	97.6%, 249/255 (94.9%–99.1%)	99.1%, 105/106 (94.9%–100.0%)	96.6%, 144/149 (92.3%–98.9%)	0.95 (0.91–0.99)
		Emergency department	100.0%, 51/51 (93.0%–100.0%)	100.0%, 13/13 (75.3%–100.0%)	100.0%, 38/38 (90.7%–100.0%)	1.00 (1.00–1.00)
	ADV	Inpatient	94.2%, 114/121 (88.4%–97.6%)	100.0%, 10/10 (69.2%– 100.0%)	93.7%, 104/111 (87.4%–97.4%)	0.71^*a*^ (0.51–0.91)
		Outpatient	95.7%, 244/255 (92.4%–97.8%)	100%, 72/72 (95.0%–100.0%)	94.0%, 172/183 (89.5%–97.0%)	0.90 (0.84–0.96)
		Emergency department	96.1%, 49/51 (86.5%–99.5%)	77.8%, 7/9^*a*^ (40.0%–97.2%)	100.0%, 42/42 (91.6%–100.0%)	0.85^*a*^ (0.65–1.00)

^*a*^ The relatively low value may be due to the small number of ADV samples for analysis.

^*b*^ The agreement parameters of the Coyote FlashDetect LyocartE assays relative to conventional Sansure real-time PCR assay are displayed by different patient types, including overall percent agreement (OPA), positive percent agreement (PPA), negative percent agreement (NPA) with 95% confidence intervals (95% CIs), and Cohen’s *κ* values. *n*/*N* = number of concordant results/total number of samples.

The Coyote FlashDetect LyocartE SARS-CoV-2/FluA/FluB assay demonstrated high concordance for SARS-CoV-2 and FluB, with all agreement metrics exceeding 93.3% and intergroup differences remaining below 4.1%. However, the PPA for FluA was lower among inpatients (84.8%, 56/66, 95% CI: 73.9%–92.5%) compared to outpatients (89.4%, 84/94, 95% CI: 81.3%–94.8%) and ED cases (89.5%, 102/114, 95% CI: 82.3%–94.4%). Parallelly, the Coyote FlashDetect LyocartE RSV/MP/ADV assay showed consistently high agreement, with all agreement metrics for RSV and MP exceeding 93.1% and intergroup differences remaining below 5.0%. In contrast, the lower PPA and reduced Cohen’s *κ* values for ADV were noted, which may be attributed to the small sample sizes, with only 10 inpatients and 9 ED cases included.

### The agreement analysis between Coyote multiplex mPOCT assays and Sansure across medical institutions of different levels

To evaluate the clinical performance of Coyote mPOCT assays for each target pathogen across hospitals of different levels, a site-level analysis was conducted (Table 6). Consistent with the findings across patient types, the Coyote assays showed good and consistent results across the four hospitals, with agreement metrics exceeding 90.0% for SARS-CoV-2, FluB, RSV, and MP, whereas relatively lower agreement was observed for FluA and ADV. For FluA, targeted by Coyote FlashDetect LyocartE SARS-CoV-2/FluA/FluB assay, significantly lower OPA and PPA values were observed in Beijing Chaoyang Hospital compared to the other three sites. Specifically, the Coyote assay showed OPA values of 83.1% (69/83, 95% CI: 73.3% to 90.5%) and PPA values of 60.0% (18/30, 95% CI: 40.6% to 77.3%), respectively. In contrast, Coyote assay showed OPA and PPA values above 90.0% in the other three hospitals. For ADV, targeted by Coyote FlashDetect LyocartE RSV/MP/ADV assay, the lower PPA at Beijing Hospital was likely attributable to the small sample size (*n* = 4), standing in contrast to the 100% PPA achieved at the other three sites.

**TABLE 6:** The agreement analysis between Coyote multiplex mPOCT assays against Sansure assay across medical institutions of different levels^*c*^

mPOCT assays	Target pathogens	Clinical sites	OPA, *n*/*N* (95% CI)	PPA, *n*/*N* (95% CI)	NPA, *n*/*N* (95% CI)
**Coyote FlashDetect** **SARS-CoV-2/FluA/FluB**	**SARS-CoV-2**	**Beijing**	92.5%, 136/147 (87.0%–96.2%)	90.7%, 68/75 (81.7%–96.2%)	94.4%, 68/72 (86.4%–98.5%)
		**Chaoyang**	98.8%, 82/83 (93.5%–100.0%)	93.8%, 15/16 (69.8%–99.8%)	100.0%, 67/67 (94.6%–100.0%)
		**Shunyi**	98.0%, 342/349 (95.9%–99.2%)	96.8%, 184/190 (93.3%–98.8%)	99.4%, 158/159 (96.5%–100.0%)
		**Fenxian**	96.8%, 61/63 (89.0%–99.6%)	100.0%, 8/8 (63.1%–100.0%)	96.4%, 53/55 (87.5%–99.6%)
	**FluA**	**Beijing**	91.8%, 135/147 (86.2%–95.7%)	92.2%, 94/102 (85.1%–96.6%)	91.1%, 41/45 (78.8%–96.6%)
		**Chaoyang**	83.1%, 69/83 (73.3%–90.5%)	60.0%, 18/30 (40.6%–77.3%)	96.2%, 51/53 (87.0%–99.5%)
		**Shunyi**	96.3%, 336/349 (93.7%–98.0%)	90.7%, 127/140 (84.6%–95.0%)	100.0%, 209/209 (98.3%–100.0%)
		**Fenxian**	98.4%, 62/63 (91.5%–100.0%)	100.0%, 2/2 (15.8%–100.0%)	98.4%, 60/61 (91.2%–100.0%)
	**FluB**	**Beijing**	100%, 147/147	NA^*a*^	100.0%, 147/147 (97.5%–100.0%)
		**Chaoyang**	100%, 83/83	NA^*a*^	100.0%, 83/83 (95.6%–100.0%)
		**Shunyi**	100%, 349/349 (98.9%–100.0%)	100%, 3/3 (92.6%–100.0%)	100.0%, 346/346 (98.9%–100.0%)
		**Fenxian**	100%, 63/63 (94.3%–100.0%)	100%, 60/60 (94.0%–100.0%)	100.0%, 3/3 (29.2%–100.0%)
**Coyote FlashDetect** **RSV/MP/ADV**	**RSV**	**Beijing**	100.0%, 33/33 (89.4%–100.0%)	100.0%, 10/10 (69.2%–100.0%)	100.0%, 23/23 (85.2%–100.0)
		**Chaoyang**	98.6%, 68/69 (92.2%–100.0%)	93.8%, 15/16 (69.8%–99.8%)	100.0%, 53/53 (93.3%–100.0%)
		**Shunyi**	98.0%, 294/300 (95.7%–99.3%)	94.4%, 84/89 (87.4%–98.2%)	99.5%, 210/211 (97.4%–100.0%)
		**Fenxian**	92.0%, 23/25 (74.0%–99.0)	91.3%, 21/23 (72.0%–98.9%)	100.0%, 2/2 (15.8%–100.0%)
	**MP**	**Beijing**	100.0%, 33/33 (89.4%–100.0%)	100.0%, 18/18 (81.5%–100.0%)	100.0%, 15/15 (78.2%–100.0%)
		**Chaoyang**	97.1%, 67/69 (89.9%–99.6%)	100%, 7/7 (59.0%–100.0%)	96.8%, 60/62 (88.8%–99.6%)
		**Shunyi**	98.3%, 295/300 (96.2%–99.5%)	98.2%, 111/113 (93.8%–99.8%)	98.4%, 184/187 (95.4%–99.7%)
		**Fenxian**	100.0%, 25/25 (86.3%–100.0%)	100.0%, 1/1 (2.5%–100.0%)	100.0%, 24/24 (85.8%–100.0%)
	**ADV**	**Beijing**	94.0%, 31/33 (79.8%–99.3%)	50.0%, 2/4^*b*^ (6.8%–93.2%)	100.0%, 29/29 (88.1%–100.0%)
		**Chaoyang**	97.1%, 67/69 (89.9%–99.6%)	100%, 7/7 (59.0%–100.0%)	96.8%, 60/62 (88.8%–99.6%)
		**Shunyi**	95.3%, 286/300 (92.3%–97.4%)	100.0%, 79/79 (95.4%–100.0%)	93.7%, 207/221 (89.6%–96.5%)
		**Fenxian**	92.0%, 23/25 (74.0%–99.0%)	100.0%, 1/1 (2.5%–100.0%)	91.7%, 22/24 (73.0%–99.0%)

^*a*^ NA, not applicable. The calculation of PPA is not applicable due to the absence of positive samples.

^*b*^ The relatively low value may be due to the small number of samples for analysis.

^*c*^ The agreement parameters of the Coyote FlashDetect LyocartE assays relative to conventional Sansure real-time PCR assay are displayed by medical institutions of different levels, including overall percent agreement (OPA), positive percent agreement (PPA), negative percent agreement (NPA) with 95% confidence intervals (95% CIs), and Cohen’s *κ* values. *n*/*N* = number of concordant results/total number of samples. Abbreviations: Beijing, Beijing Hospital; Chaoyang, Beijing Chaoyang Hospital; Shunyi, Beijing Shunyi Hospital; Fenxian, Shanghai Fenxian Central Hospital.

### The target-specific paired comparison between Coyote and Cepheid multiplex mPOCT assays for FluA, FluB, and RSV

We directly evaluated the performance of the Coyote assays by comparing them against the internationally recognized Cepheid Xpert Xpress Flu/RSV assay for FluA, FluB, and RSV detection, and the specific data were displayed in Table 7 by pathogen target. For FluA and FluB, the Coyote FlashDetect LyocartE SARS-CoV-2/FluA/FluB assay demonstrated excellent consistency with Cepheid Xpert Xpress Flu/RSV, achieving the PPA of 96.0% (238/248, 95% CI: 92.7% to 98.1%) for FluA and 100% (61/61, 95% CI: 94.1% to 100.0%) for FluB, as well as the NPA of >96.0% for both viruses. For RSV, the Coyote FlashDetect LyocartE RSV/MP/ADV assay achieved a high PPA of 98.3% (115/117, 95% CI: 94.0% to 99.8%) although the NPA was comparatively lower at 83.7% (82/98, 95% CI: 74.8% to 90.4%). The results of the Ct-stratified agreement analysis demonstrated that PPA values were above 99.0% for FluA, FluB, and RSV among samples with high viral loads (Ct < 35), whereas reduced PPA values were observed in samples with higher Ct values, ranging from 81.4% to 100% (Table 4). For the correlation analysis of Ct values in positive samples, strong correlations were observed for FluA (*r* = 0.96, 95% CI: 0.94 to 0.97), FluB (*r* = 0.96, 95% CI: 0.94 to 0.98), and RSV (*r* = 0.90, 95% CI: 0.86–0.93) (Fig. 3A through C).

**TABLE 7:** The agreement analysis between the Coyote FlashDetect LyocartE and the Cepheid assays^*a*^

Coyote FlashDetect LyocartE			Cepheid Xpert Xpress Flu/RSV		OPA, *n/N* (95% CI)	PPA,*n/N* (95% CI)	NPA, *n/N* (95% CI)	Cohen’s *κ* (95% CI)
			Positive	Negative				
Coyote FlashDetect SARS-CoV-2/FluA/FluB	FluA	Positive	238	6	96.1%, 398/414 (93.8%–97.8%)	96.0%, 238/248 (92.7%–98.1%)	96.4%, 160/166 (92.3%–98.7%)	0.92 (0.88–0.96
		Negative	10	160				
		Total	248	166				
	FluB	Positive	61	2	99.5%, 412/414 (98.3%–99.9%)	100.0%, 61/61 (94.1%–100.0%)	99.4%, 351/353 (98.0%–99.9%)	0.98 (0.95–1.00)
		Negative	0	351				
		Total	61	353				
Coyote FlashDetect RSV/MP/ADV	RSV	Positive	115	16	91.6%, 197/215 (87.1%–95.0%)	98.3%, 115/117 (94.0%–99.8%)	83.7%, 82/98 (74.8%–90.4%)	0.83 (0.75–0.90)
		Negative	2	82				
		Total	117	98				

^*a*^ Note: The agreement parameters of the Cepheid Xpert Xpress and the Coyote FlashDetect LyocartE assays are displayed by pathogen type, including overall percent agreement (OPA), positive percent agreement (PPA), negative percent agreement (NPA) with 95% confidence intervals (95% CIs), and Cohen’s *κ* values. *n/N* = number of concordant results/total number of samples.

A total of 36 discrepancies were observed between Coyote and Cepheid, comprising 16 for FluA, 2 for FluB, and 18 for RSV. The detailed discrepant results between coyote and Cepheid were displayed in Table 8. In general, Cepheid detected slightly more specimens classified as FluA-positive by Sansure comparator than Coyote. Among the 16 discrepant FluA results, the majority were negative discordant results relative to both comparators (62.5%, 10/16), while 3 were positive discordant against both comparators and 3 were positive discordant relative to Cepheid only. In contrast, the Coyote assay identified 15 specimens classified as RSV-positive by the Sansure comparator, which were missed by Cepheid, accounting for 83.3% (15/18) of the total RSV inter-platform discrepancies, albeit with 2 negative discordant and 1 positive discordant results against both comparators. Additionally, all discrepant FluB results were FluB-positive by the Sansure comparator but were not detected by the Cepheid assay. Similarly, high Ct values were observed in these discrepancies: 33.3–39.7 for FluA, 37.6–37.9 for FluB, and 29.2–39.8 for RSV.

**TABLE 8:** Detailed analysis of discrepant results between Cepheid and Coyote

Target pathogens	Sansure results	Cepheid results	Coyote results	Sample number	Ct values [average, min, max]	Comments
FluA *N* = 16	+	−	+	3	[37.5, 35.5, 39.7]	All samples have Ct values > 35
		+	−	10	[36.3, 33.3, 38.0]	Two samples with Ct values ≤ 35 (Ct = 33.3 and 34.1)
	−	−	+	3	[37.9, 36.9, 39.2]	All samples have Ct values > 35
FluB *N* = 2	+	−	+	2	[37.7, 37.6, 37.9]	All samples have Ct values > 35
RSV *N* = 18	+	−	+	15	[35.7, 29.2, 38.9]	Six sample with Ct value ≤ 35 (Ct = 29.2, 31.3, 33.2, 33.9, 34.4, 34.8)
		+	−	2	[35.8, 35.6, 36.0]	All samples have Ct values > 35
	−	**−**	+	1	[39.8, 39.8, 39.8]	All samples have Ct values > 35

## DISCUSSION

Nowadays, the growing demand for rapid and accurate detection of infectious pathogens in high-risk population and resource-limited areas has driven the development of multiplex mPOCT platforms. The large-scale deployment of these newly developed platforms requires thorough clinical evaluation. In this study, we conducted a multicenter evaluation of the newly certified Coyote FlashDetect LyocartE assays in comparison with the conventional laboratory-based Sansure real-time PCR assay and the internationally recognized Cepheid Xpert Xpress mPOCT assay.

Compared with the Sansure assay, the Coyote FlashDetect LyocartE mPOCT platform demonstrated high concordance, achieving an OPA of ≥93.8% for each of six pathogens (Table 3). However, the PPA of FluA (88.0%, 95% CI: 83.5% to 91.6%) was markedly lower than the other five pathogens (Table 3). After examining Ct values of FluA-positive specimens measured by the Sansure assay, we found that 21.2% (58/274) had high Ct values (Ct ≥ 35), of which 50.0% (29/58) were missed by the Coyote assays. Moreover, among the discrepant specimens between Sansure and Coyote, FluA negative discordant results accounted for 55.9% (33/59) of all negative results. And high-Ct FluA samples (Ct ≥ 35) accounted for 87.9% (29/33) of the total negative discordant FluA results from the Coyote assay (Table 4). One possible explanation is that these specimens may have Ct values near or below the analytical detection limit of the Coyote system. This is the common limitation in mPOCT platforms, and the previous study demonstrated that conventional real-time PCR had approximately 10 to 100 times lower LOD (34), and mPOCT platforms exhibit a 10%–30% reduction in clinical sensitivity when detecting high-Ct samples (34–36). However, the inferior detection performance of mPOCT platforms in high-Ct sample has lower clinical significance since the Ct values of symptomatic population are often below 35 (37, 38). Considering this, we further assessed the detection agreement of Coyote assay in 216 FluA-positive samples with Ct < 35. A PPA of 98.15% (212/216) was achieved, indicating their adequate clinical utility for detecting patients in the acute phase of infection. For different patient types, Coyote multiplex mPOCT assays showed higher agreement in FluA detection for outpatients and ED cases (Table 5). The analysis of Ct values of FluA-positive samples from the Sansure assay revealed that 27.3% (18/66) of inpatient samples had Ct values ≥ 35, higher than that of outpatients (17.0%, 16/94) and ED cases (21.1%, 24/114). These findings suggested that the higher proportion of high Ct samples in inpatients may partly explain the lower positive agreement observed in this subgroup. This difference is relative to the characteristics of the patient types. Outpatients and ED patients are always in the early stages of infection, with more self-reported symptoms and higher viral loads, facilitating more accurate detection (39). In contrast, inpatients often have more complex clinical conditions or delays in medical care and testing (40, 41). Therefore, mPOCT assays provide sufficient detection capability for use in outpatient and ED settings. And negative mPOCT results should be interpreted with caution for inpatients, with resampling or retesting with conventional real-time PCR if necessary.

Compared to other hospitals, the Coyote multiplex mPOCT assays exhibited lower PPA values for FluA detection in Beijing Chaoyang Hospital. After tracing the Ct values obtained from the Sansure assay for the 30 FluA-positive specimens from Beijing Chaoyang Hospital, we found that 19 samples had high Ct values (Ct ≥ 35), ranging from 35.4 to 40.0 (mean 37.4 ± 1.4). For these 19 samples, the Coyote assay produced PPA values of 36.8% (7/19), with 12 negative discordant results. While for the remaining 11 samples (Ct < 35), the Coyote assays achieved a PPA of 100%. This may be associated with the characteristics of the patient population. As Beijing Chaoyang Hospital conducts FluA testing every 2 days, the longer TAT could not meet the demands of patients with acute symptoms who require timely diagnosis and intervention. Thus, the population tested in Beijing Chaoyang Hospital may mainly consist of patients with mild symptoms or in the late period of infection with lower viral loads.

As an internationally recognized and widely adopted mPOCT platform, the robust performance of the Cepheid Xpert Xpress assay has been fully evaluated (42–45). Newly developed multiplex mPOCT platforms, such as the SD BioSensor STANDARD M10 Flu/RSV/SARS-CoV-2 (46), BD SARS-CoV-2/Flu (47), Cobas Liat system (48, 49), and Abbott ID Now (50), were usually benchmarked against the Cepheid assay and showed comparable or lower agreement in certain targets. In this study, the Coyote FlashDetect LyocartE assays showed high concordance with the Cepheid assay in FluA and FluB detection, with OPA ≥96.1% and Cohen’s *κ* values over 0.92. For RSV, a lower OPA of 91.6% (197/215, 95% CI: 87.1% to 95.0%), NPA of 83.7% (82/98, 95% CI: 74.8% to 90.4%), and Cohen’s *κ* value of 0.83 were observed. This may be attributed to 15 specimens classified as RSV-positive by the Sansure comparator, which were also RSV-positive by the Coyote assay but RSV-negative by the Cepheid assay, accounting for 83.3% (15/18) of the total RSV inter-platform discrepancies, among which six sample had Ct values < 35 (Table 8). Consistent with our findings, previous studies also reported that the Cepheid Xpert Xpress Flu/RSV assay demonstrated a lower PPA of 77.8% (51) and a sensitivity of 86.0% (95% CI: 76.9%–92.6%) (52) for RSV detection. However, in our study, these discordant results were not adjudicated using a third reference method and it cannot be definitively determined which assay more accurately reflects the true infection status. The factors contributing to the observed discordance remain uncertain and may be associated with differences in analytical sensitivity, viral load distribution, or other assay-related factors. One possible hypothesis is that differences between locally epidemic RSV strains in China and the reference strains used for Cepheid assay development (RSV A/2/Australia/61, RSV A/Long/MD/56, RSV B/Wash/18537/62, and RSV B/9320/MA/77) may contribute to the observed discordance (53). This phenomenon aligns with previous findings showing that locally developed reagents often perform better on local samples (54). Further investigations, including sequence analysis of discordant specimens, would be required to clarify the underlying causes.

There are several limitations in this study. First, due to the retrospective SOC-based specimen selection strategy, the study cohort may not accurately reflect the distribution of cases encountered in routine clinical practice. As a result, the enrichment of SOC-confirmed specimens can potentially inflate agreement metrics and limit generalizability to consecutively enrolled populations. Therefore, the reported agreement estimates herein should be interpreted in light of this SOC-based retrospective design and may not fully generalize to unselected consecutive populations. Second, due to the constrained prevalence, the limited number of FluB and ADV positive samples was collected, which may affect the representativeness of the clinical performance evaluation for these targets. Third, since the specimens were collected during the FluA epidemic periods, conducting FluA subtyping would be valuable for comprehensive assessment of the detection capability of the Coyote assay in subtype detection. Fourth, given the high Ct values observed in discordant specimens, discordant results between Coyote and comparator assays were not adjudicated by an independent reference method, as such samples are inherently challenging to adjudicate. Consequently, it is not possible to determine which assay result more closely reflects the true infection status for any given discordant specimens. Studies using advanced molecular methods with higher sensitivity are required to resolve these differences.

In conclusion, the Coyote FlashDetect LyocartE assays achieved high concordance with the Sansure assays for these six pathogens across the full study cohort. In the target-specific subset analysis, Coyote showed comparable agreement with Cepheid for shared targets although this comparison was limited to pre-allocated specimens rather than a full head-to-head evaluation across all samples. Notably, while positive agreement declined for specimens with high Ct values (Ct ≥ 35), Coyote showed strong positive agreement with Sansure for the majority of acute-phase clinical specimens (Ct < 35). These findings suggested that the Coyote mPOCT assay may serve as a viable, rapid, and user-friendly alternative for multiplex respiratory pathogen detection and holds the promise to be used in primary healthcare settings and public health surveillance.

## Data Availability

The data supporting the findings of this study are available in the main text. No new sequence data were generated. The detailed metadata for each sample are available from the corresponding author upon reasonable request.
